# Supplementary figures and images for: Analysis of the Effects of Ninjin’yoeito on Physical Frailty in Mice
Source: Int J Mol Sci. 2022 Sep 23;23(19):11183. doi: 10.3390/ijms231911183 (PMC9569708; doi:10.3390/ijms231911183)

Figure S1.

**A**

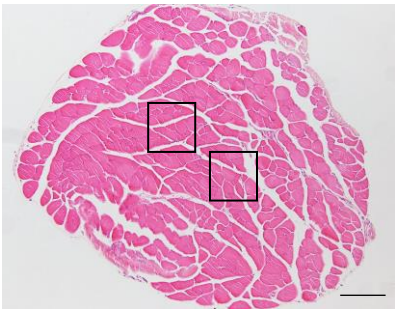

**B**

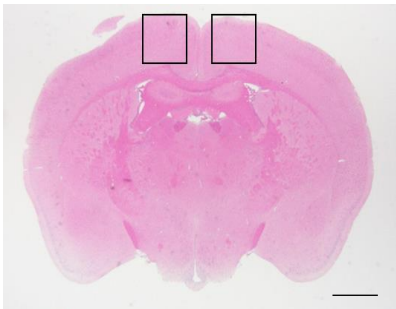

Supplement: Supplementary file 1 [file ijms-23-11183-s001.zip › ijms-1865071-supplementary.pdf]
